# Proteomic Analysis of *Trichinella spiralis* Adult Worm Excretory-Secretory Proteins Recognized by Sera of Patients with Early Trichinellosis

**DOI:** 10.3389/fmicb.2017.00986

**Published:** 2017-05-31

**Authors:** Zhong Q. Wang, Ruo D. Liu, Ge G. Sun, Yan Y. Song, Peng Jiang, Xi Zhang, Jing Cui

**Affiliations:** Department of Parasitology, Medical College, Zhengzhou UniversityZhengzhou, China

**Keywords:** *Trichinella spiralis*, trichinellosis, adult worms, excretory-secretory antigens, immunoproteomics, early diagnosis

## Abstract

The most commonly used serodiagnostic antigens for trichinellosis are the excretory-secretory (ES) antigens from *T. spiralis* muscle larvae (ML), but the specific antibodies against the ML ES antigens are usually negative during early stage of *Trichinella* infection. The recent studies demonstrated that *T. spiralis* adult worm (AW) antigens were recognized by mouse or swine infection sera on Western blot as early as 7–15 days post-infection (dpi), the AW antigens might contain the early diagnostic markers for trichinellosis. The purpose of this study was to screen early diagnostic antigens in *T. spiralis* AW ES proteins recognized by sera of early patients with trichinellosis. *T. spiralis* AW were collected at 72 h post-infection (hpi), and their ES antigens were analyzed by SDS-PAGE and Western blot. Our results showed that 5 protein bands (55, 48–50, 45, 44, and 36 kDa) were recognized by sera of early patients with trichinellosis collected at 19 dpi, and were subjected to shotgun LC–MS/MS and bioinformatics analyses. A total of 185 proteins were identified from *T. spiralis* protein database, of which 116 (67.2%) proteins had molecular weights of 30∼60 kDa, and 125 (67.6%) proteins with pI 4–7. Bioinformatic analyses showed that the identified proteins have a wide diversity of biological functions (binding of nucleotides, proteins, ions, carbohydrates, and lipids; hydrolase, transferase, and oxidoreductase, etc.). Several enzymes (e.g., adult-specific DNase II, serine protease and serine protease inhibitor) could be the invasion-related proteins and early diagnostic markers for trichinellosis. Moreover, recombinant *T. spiralis* serine protease (rTsSP-ZH68) was expressed in *E. coli* and its antigenicity was analyzed by Western blot with the early infection sera. The rTsSP-ZH68 was recognized by sera of infected mice at 8–10 dpi and sera of early patients with trichinellosis at 19 dpi. *T. spiralis* AW proteins identified in this study, especially serine protease, are the promising early diagnostic antigens and vaccine candidates for trichinellosis.

## Introduction

*Trichinella spiralis*, an intracellular parasitic nematode, can cause a serious foodborne trichinellosis. Humans acquire trichinellosis by ingesting raw or undercooked meat (mainly pork) that contains the infective larvae of *Trichinella* (Murrell, 2013). Human trichinellosis has been reported in 55 countries of the world and is considered as a re-emerging disease (Pozio, 2007). From 1986 to 2009, there were 65,818 cases and 42 deaths reported from 41 countries (Murrell and Pozio, 2011). In China, 17 outbreaks of human trichinellosis consisting of 828 cases were reported from 2000 to 2003, and 15 outbreaks of trichinellosis consisting of 1387 cases and 4 deaths occurred during 2004–2009 (Wang et al., 2006; Cui et al., 2011). Trichinellosis can lead to death, particularly in elderly patients accompany with neurological or cardiovascular complications. However, it is difficult to diagnose the human trichinellosis on the basis of clinical manifestations of the patients. (Dupouy-Camet et al., 2002).

*Trichinella spiralis* muscle larvae (ML) excretory-secretory (ES) antigens, recommended by the International Commission on Trichinellosis (ICT), are the most commonly used serodiagnostic antigens for trichinellosis (Gamble et al., 2004), but the specific antibodies against the ML ES antigens are usually negative during early stage of *Trichinella* infection (Cui et al., 2015), possibly because the majority of ML ES antigens are the stage-specific and not recognized by specific antibodies produced during the intestinal phase (Liu J.Y. et al., 2016). Previous studies have shown that 100% detection of anti-*Trichinella* IgG is not possible for at least 1–3 months after primary *Trichinella* infection (Bruschi et al., 2001). There is an obvious window period of 3–4 weeks between *Trichinella* infection and specific antibody positivity.

After being ingested, *T. spiralis* ML develop to intestinal infective larvae which invade the host’s small intestinal epithelium, and undergo four molting to develop to adult worms (AW) in 31 h post-infection (hpi). After mating, the female AW invade intestinal mucosa again and live there for 10–20 days in mice and rats or 4–6 weeks in human (Campbell, 1983). During the intestinal stage of *T. spiralis* infection, the ES antigens produced by the AW result in early exposure to the host’s immune system and elicit the production of specific anti-*Trichinella* antibodies. The ES proteins of intestinal AW of *T. spiralis* might contain the early diagnostic markers of trichinellosis (Wang et al., 2017). The immunoproteomics study showed that 64 proteins in AW crude extracts were recognized by sera from *T. spiralis*-infected pig and mice at 7 days post-infection (dpi), and the recombinant Ts14-3-3 had the significant potential as early diagnostic reagents or vaccine candidates (Yang et al., 2015). The recombinant protein from pre-adults 20 hpi was recognized by swine infection sera on Western blot as early as 15–20 dpi (Zocevic et al., 2014). In our previous studies, anti-*Trichinella* IgG of infected mice at 8 dpi was detected by ELISA using AW ES antigens, while anti-*Trichinella* IgG cannot be detected before 12 dpi; meanwhile the detection of anti-*Trichinella* IgG of patients with trichinellosis at 19 dpi demonstrated that the sensitivity of AW ES antigens (100%) were superior to ML ES antigens (75%) (Sun et al., 2015). Additionally, one protein band with 33 kDa in *T. spiralis* AW ES antigens was recognized by sera of infected mice at 8 dpi and 10 proteins of *T. spiralis* were identified by mass spectrometry (Liu R.D. et al., 2016). However, *T. spiralis* AW ES antigens recognized by sera of patients with trichinellosis have not yet been explored and they may contain the key target antigens important for host’s immune recognition and diagnostic markers for early detection of *Trichinella* infection in humans. Therefore, it is possible to screen and characterize the new early diagnostic antigens from *T. spiralis* AW.

In this study, *T. spiralis* AW ES antigens were separated by SDS-PAGE and recognized by early sera of patients with trichinellosis in Western blot, then the recognized bands were selected for protein identification by shotgun LC-MS/MS analyses in combination with bioinformatics. These data are expected to provide valuable information for early diagnostic antigens for trichinellosis.

## Materials and Methods

### Ethics Statement

This study was carried out in accordance with the National Guidelines for Experimental Animal Welfare (MOST of People’s Republic of China, 2006). The protocol was approved by The Life Science Ethics Committee of Zhengzhou University. All individuals give oral informed consent before the use of serum samples from healthy donors and patients with trichinellosis.

### Parasites and Animals

The *T. spiralis* isolate (ISS534) used in the present study was obtained from domestic pigs in Nanyang city of Henan Province, China. The *Trichinella* isolate was kept by the serial passage in BALB/c mice every 6–8 months in our laboratory. Specific pathogen-free (SPF) female BALB/c mice and Wistar rats were purchased from the Henan Provincial Experimental Animal Centre (Zhengzhou, China).

### Collection of *T. spiralis* AW and Preparation of AW ES Antigens

*Trichinella spiralis* ML were collected from the skeletal muscles of infected mice at 42 dpi with an artificial digestion method (Gamble et al., 2000; Li et al., 2010). After recovery, the ML were used to orally inoculate 30 rats at 8,000 ML per rat. The AW were collected from the upper two-thirds (duodenum and jejunum) of small intestine of experimentally infected rats at 72 hpi (Sun et al., 2015). The collected AW were washed five times in PBS with 100 U penicillin/ml and 100 μg streptomycin/ml and then cultured at 37°C in RPMI 1640 and 5% CO_2_ for 18 h. After incubation, The supernatant contained ES proteins was obtained by centrifugation at 4°C, 11,000 *g* for 20 min, and concentrated using an Amicon Ultra-3 Centrifugal Filter Unit (molecular weight [MW] cut-off, 3 kDa; Millipore, United States) at 4°C and 5, 000 *g* for 1 h (Liu R.D. et al., 2016). The ES antigen concentration was determined using the Bradford method and stored at -80°C before use.

### Serum Samples

Serum samples from patients with trichinellosis were collected from the patients during two outbreaks with trichinellosis, which occurred in the Yunnan province located in southwestern China in 2003 (10 serum samples collected at 19 dpi) and 2013 (3 serum samples collected at 35 dpi) (Wang et al., 2006; Wang C.Q. et al., 2013). These patients had a history of ingestion of raw or undercooked meat and presented with typical clinical manifestations of trichinellosis (fever related with periorbital or facial oedema, myalgia, and eosinophilia). All these patients had serum specific anti-*Trichinella* IgG antibody by ELISA with ML ES antigens (Cui et al., 2015; Sun et al., 2015). The optical density values at 490 nm of ten serum samples from patients with early trichinellosis at 19 dpi were 0.54, 0.535, 0.585, 0.615, 0.545, 0.585, 0.56, 0.59, 0.53, and 0.575, respectively; the cut-off value was 0.45. Serum samples from presumably healthy persons, who tested negative for anti-*Trichinella* IgG, were also included in the study. All the serum samples were stored at -80°C until used.

The mouse infection sera were collected from 10 experimentally infected mice at 42 dpi, each mouse was orally infected with 100 *T. spiralis* ML. Sera from uninfected mice were collected as negative controls and sera collected from the mice infected with *T. spiralis* at 42 dpi were used as positive controls.

### SDS-PAGE and Western Blot Analysis

The AW ES antigens (20 μg) were subjected to sodium dodecyl sulfat-polyacrylamide gel (SDS-PAGE) in 5% stacking gels and 12% resolving gels at 80 V for 40 min and 120 V for 90 min. After electrophoresis, one gel was stained in Coomassie brilliant blue R-250 staining solution (Sigma, United States) for 4 h, and the other gel was used for the electrotransfer of proteins on nitrocellulose (NC) membranes (Millipore, United States) at 18 V for 35 min via a semi-dry transfer cell (Bio-Rad, United States) (Wang B. et al., 2013). Subsequently, the membranes were cut into strips, blocked with 5% skim milk in TBST (20 mM Tris-HCl, 150 mM NaCl, 0.05% Tween-20, pH 7.6) at 37°C for 1 h, and incubated at 4°C overnight with sera from different patients with trichinellosis at a dilution of 1:100. After being washed in TBST, the strips were incubated with HRP-conjugated goat anti-human IgG (1:5,000 dilutions; Sigma, United States) at 37°C for 1 h. Finally, the immunoreaction was detected with 3, 3-diaminobenzidine tetrahydrochloride (DAB; Sigma, United States). The gel and membranes were scanned using ImageScanner (GE healthcare, United States) and the MW of these bands was analyzed by AlphaView software (ProteinSimple, Santa Clara, CA, United States).

### Mass Spectrometry Analysis

Protein bands recognized by sera of early patients with trichinellosis were excised from the parallel gel and subjected to in-gel tryptic digestion according to an optimized procedure (Wang et al., 2012). Briefly, the gel was washed two times, 10 min each, with Milli-Q water and then destained with 25 mM NH_4_HCO_3_ in 50% acetonitrile at 37°C until depigmentation. Then, the dried gel was incubated with 10 mM dithiothreitol at 56°C for 1 h and alkylated with 55 mM iodoacetamide at room temperature for 45 min in the dark. Gel pieces were washed two times with 100 μL of 25 mM NH_4_HCO_3_ for 10 min, destained as above for 10 min and then dehydrated in a vacuum centrifuge. Subsequently, the gel pieces were digested in 20 ng/μl trypsin buffers at 37°C overnight, followed by dissolved in 0.1% formic acid for shotgun analysis. Then, the proteins were separated by high performance liquid chromatography and analyzed by tandem MS, as previously described (Liu et al., 2010). Peptides were ionized in positive ion mode and introduced into the Q-Exactive Orbitrap mass spectrometer (Thermo Fisher Scientific, Waltham, MA, United States). Initial separation was performed on a PepMap C18 reverse phase capillary column (3 μm Hypersil C18, 75 μm × 2cm, Thermo Fisher Scientific, Waltham, MA, United States). Mobile phases were 0.1% FA in Millipore water as buffer A and 0.1% FA in 84% ACN as buffer B. Peptides were eluted using a 65 min gradient of 5–80% buffer B at a flow rate of 400 nL/min. The MS/MS data were searched against the *T. spiralis* protein database from UniProt and NCBI using the SEQUEST algorithm. The SEQUEST search parameters used in this study were Delta CN (≥0.1) and Xcorr (one charge ≥ 1.9, two charges ≥ 2.2, and three charges ≥ 3.75) (Liu et al., 2015).

### Bioinformatics Analysis

The signal peptide and transmembrane domains of the identified proteins were predicted by using online tool SignalP^1^, TMHMM^2^. To assign a possible function, gene ontology (GO) features of the identified proteins were obtained using Uniprot and InterPro databases (Mitchell et al., 2015). Then, the Web Gene Ontology Annotation Plotting (WEGO^3^) was used to analysis the GO categories (Ye et al., 2006).

### Expression and Immunological Test of Recombinant *T. spiralis* Serine Protease (rTsSP-ZH68)

*Trichinella spiralis* TsSP-ZH68 gene was amplified by PCR using specific primers with BamHI and Pst1 restriction enzyme sites (5′-TTCGGATCCAATTATGAATGTG GCACCTTAC-3′ and 5′-CCGCTGCAGTTAACGGAAAAAAGTGAATGAT-3′). The amplified PCR products were purified, digested, and cloned into the pGEM-T vector (Promega, United States) and subsequently sub-cloned into the expression vector PQE-80L (New England Biolabs, United States). The recombinant plasmid PQE-80L/TsSP-ZH68 was transformed into the *Escherichia coli* BL21. The rTsSP-ZH68 was expressed and identified in our laboratory (unpublished data). The purified rTsSP-ZH68 was identified by SDS-PAGE and transferred onto the NC membrane for Western blot analysis. The membrane was incubated with sera of the infected mice or sera of patients with trichinellosis to identify the immunogenic antigens.

## Results

### SDS-PAGE and Western Blotting Analysis

**FIGURE 1:**
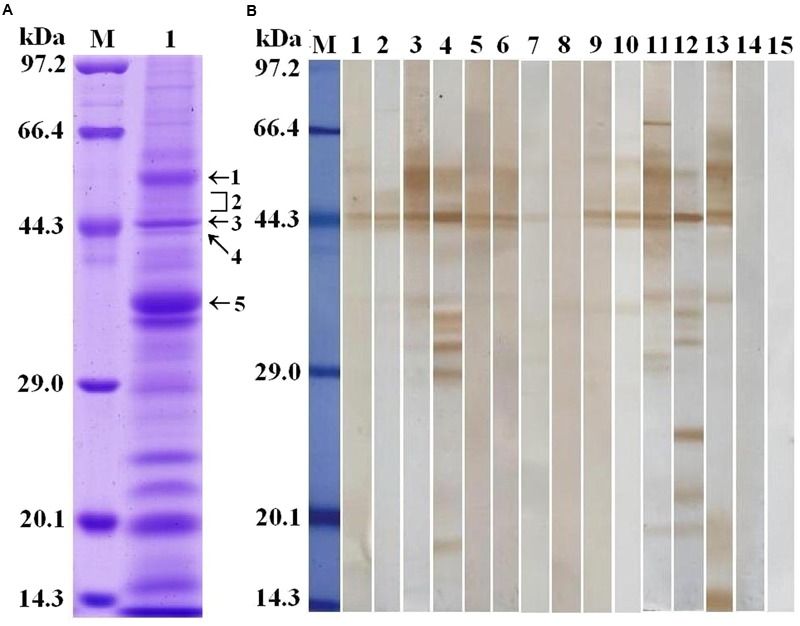
Analysis of *T. spiralis* AW ES proteins recognized by sera of early patients with trichinellosis. **(A)** SDS-PAGE analysis of *T. spiralis* AW ES proteins. AW ES proteins were separated on 12% polyacrylamide gels. Lane M = low molecular weight protein marker; Lane 1 = AW ES proteins. The arrows indicate the 5 protein bands (55, 48–50, 45, 44, and 36 kDa) which were excised, digested, and analyzed by LC–MS/MS. **(B)** Western blotting of AW ES proteins probed by sera of early patients with trichinellosis. Lane M = low molecular weight protein marker; Lane1-10 = anti-*Trichinella* IgG-positive sera from early patients with trichinellosis at 19 dpi; Lane 11–13 = anti-*Trichinella* IgG-positive sera from patients with trichinellosis at 35 dpi; Lane 14–15 = sera from healthy persons.

*Trichinella spiralis* AW ES proteins were separated by SDS-PAGE. The results demonstrated that the AW ES proteins had 25 bands with a MW ranging from 15 to 97 kDa (**Figure 1A**). The results of Western blot analysis indicated that fifteen protein bands (77, 61, 55, 50, 48, 45, 44, 42, 36, 35, 33, 32, 29, 27, and 17 kDa) were recognized by sera of early patients with trichinellosis at 19 dpi, and sixteen protein bands (77, 61, 55, 50, 48, 45, 44, 42, 36, 35, 33, 32, 29, 24, 20, and 17 kDa) were recognized by sera of patients with trichinellosis at 35 dpi (**Figure 1B**). But, sera from healthy persons could not recognize these protein bands. The five protein bands (55, 48–50, 45, 44, and 36 kDa) recognized by eight serum sample of early patients with trichinellosis were, respectively, used to the shotgun LC-MS/MS analysis.

### Protein Identification by Shotgun LC-MS/MS Analysis

**FIGURE 2:**
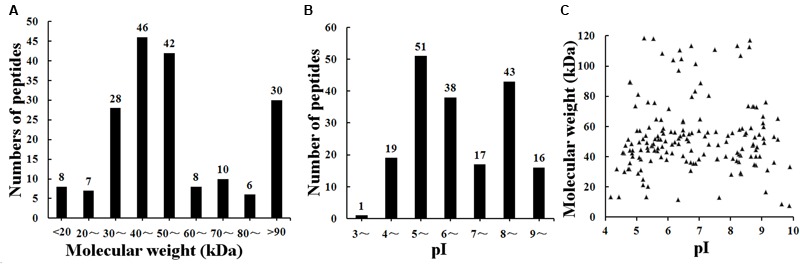
Distribution of identified 185 *T. spiralis* proteins of AW ES proteins recognized by sera of early patients with trichinellosis collected at 19 dpi. **(A)** Molecular weight (MW). **(B)** Isoelectric point (pI). **(C)** Two-dimensional (MW and pI).

The result of shotgun LC-MS/MS analysis showed that total of 185 *T. spiralis* proteins with unique pep counts ≥ 2 were identified from *T. spiralis* protein database, of which 133 (71.89%) proteins were annotated by InterProscan software. Several enzymes (e.g., adult-specific DNase II, serine protease and serine protease inhibitor) might be the invasion-related proteins and early diagnostic antigens for trichinellosis, and part of the proteins identified were shown in **Table 1**. The MW of 185 proteins varied from 7.38 to 493.65 kDa with 116 (62.70%) proteins distributed in the range of 30∼60 kDa (**Figure 2**). The pI value ranged from 3.98 to 9.90, with 80.54% of proteins distributed between 5 and 9. Out of 185 proteins, 48 (25.95%) proteins had signal peptide and 37 (20.00%) had a transmembrane domain (Supplementary Table 1).

**Table 1:** A part of the identified ES proteins of *T. spiralis* AW recognized by sera of early patients with trichinellosis collected at 19 dpi.

Protein name	Uniprot ID	Theoretical Mr/pI	Cover percent (%)	From protein bands (kDa)	Signal peptide	*Trans*- Membrane domain	Function
Putative nudix hydrolase	B0F9S5	46.51/8.85	76	55	No	Yes	Hydrolase activity
Chymotrypsin-like protease	A0A0F6UUK4	31.68/4.76	26	45, 44	Yes	No	peptidase activity
Histone deacetylase 1	A0A0V0W9U4	35.43/6.88	23	45	No	No	Histone deacetylase activity;hydrolase activity
Enteropeptidase	A0A0V0X5K0	75.83/5.57	9.9	36	Yes	No	serine-type endopeptidase activity
Adenylosuccinate lyase	A0A0V1ASK3	186.79/6.37	16.8	55, 48–50, 45	No	No	Catalytic activity;N6-(1,2-dicarboxyethyl)AMP AMP-lyase (fumarate-forming) activity;GTPase activator activity;lyase activity
Phosphotransferase	A0A0V1B3W4	51.73/5.59	68.8	48–50	No	No	Nucleotide binding;hexokinase activity;ATP binding;glucose binding;kinase activity;transferase activity;phosphotransferase activity, alcohol group as acceptor
Alpha-(1,3)-fucosyltransferase C	A0A0V1BJB0	43.11/5.96	73.1	55, 48–50, 45, 44	Yes	No	Fucosyltransferase activity;
Histone deacetylase	A0A0V1BMM5	54.05/5.30	17.9	55	No	No	Histone deacetylase activity
Fumarate hydratase, class II	E5S0V2	54.72/8.46	56.2	48–50	No	No	Catalytic activity; fumarate hydratase activity; lyase activity
Serine protease inhibitor family protein	E5SCW1	39.73/5.78	40.7	48–50, 45, 44	No	No	Peptidase activity
Enteropeptidase	E5SUQ1	76.03/5.37	21.9	55, 48–50	Yes	No	Serine-type endopeptidase activity
Phosphoglycerate kinase	E5SVP3	59.92/9.06	65.9	44	No	Yes	Phosphoglycerate kinase activity;
Adult-specific DNase II-7	Q32R69	38.54/7.56	37.4	48–50	Yes	No	Deoxyribonuclease II activity
Serine protease	Q9BJM1	72.78/8.83	66.3	55, 48–50, 45, 44, 36	Yes	No	Serine-type endopeptidase activity;
Putative serine protease	B0F9T9	48.15/8.73	30.5	55, 48–50, 45, 44	Yes	No	Serine-type endopeptidase activity

### Function Assignment in Identified Proteins through Gene Ontology

**FIGURE 3:**
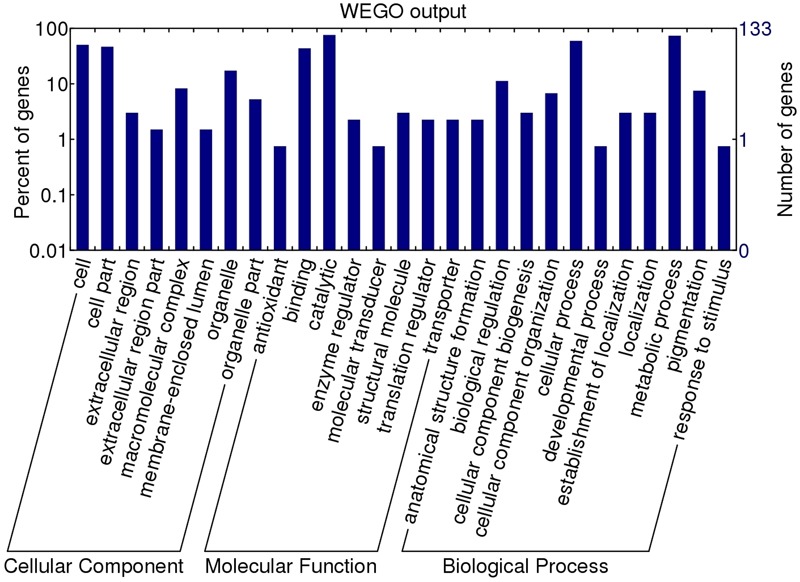
Gene ontology categories of the identified *T. spiralis* AW ES proteins recognized by sera of early patients with trichinellosis collected at 19 dpi. The proteins were grouped into cellular component, molecular function, and biological process in accordance with their GO signatures. The right-hand showed the number of proteins with GO annotations. The percent of genes denotes that the proportion of GO terms in total genes.

To assign a possible function, the GO features of the 133 proteins with GO annotation were obtained by using Uniprot and InterProscan databases. The WEGO tool was applied to plot the GO annotation distribution (**Figure 3**). These proteins were grouped into three hierarchically structured GO terms, namely biological process, cellular component, and molecular function. The main biological process of these proteins was metabolic process (98 proteins, 73.68%) and cellular process (79 proteins, 59.40%). In the cellular component ontology, a large proportion of proteins were related to cell part (62 proteins, 46.62%) and intracellular (44 proteins, 33.08%). For molecular function category, catalytic activity (101 proteins, 75.94%) and binding (58 proteins, 43.61%) were the two main molecular function categories. The assigned catalytic activity of the 101 proteins could be assigned to hydrolase, transferase, oxidoreductase, isomerase, ligase, and lyase activity. This analysis indicated that the identified proteins involved in these GO categories might play the important roles in larval invasion of host intestinal epithelium cells (IECs) and immune escape.

### Recognition of rTsSP-ZH68 by Early Infection Sera

**FIGURE 4:**
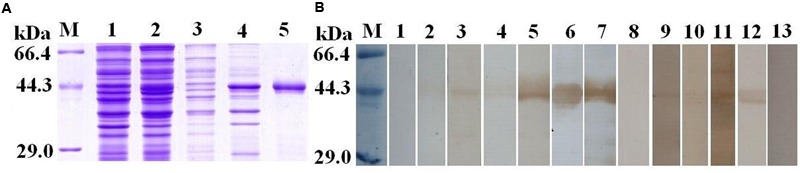
Sodium dodecyl sulfat-polyacrylamide gel and Western blot analysis of rTsSP-ZH68 antigenicity. **(A)** SDS–PAGE analysis of rTsSP-ZH68. Lane M = low molecular weight protein marker; Lane 1 = non-induced recombinant bacteria lysate; Lane 2 = IPTG-induced recombinant bacteria lysate; Lane 3 = soluble fraction of induced recombinant bacteria; Lane 4 = insoluble fraction of induced recombinant bacteria; Lane 5 = purified rTsSP-ZH68. **(B)** Western blot analysis of rTsSP-ZH68 antigenicity. Lane 1–4 = sera of mice infected with *T. spiralis* at 7–10 dpi; Lane 5 = sera of mice infected with *T. spiralis* at 15 dpi; Lane 6 = sera of mice infected with T. spiralis at 42 dpi; Lane 7 = anti-His tag mouse monoclonal antibody; Lane 8 = sera of normal mice; Lane 9–10 = anti-*Trichinella* IgG- positive sera from early patients with trichinellosis at 19 dpi; Lane 11–12 = anti-*Trichinella* IgG-positive sera from patients with trichinellosis at 35 dpi; Lane 13 = sera from healthy persons.

To validate the potential of TsSP-ZH68 as early diagnostic antigen or vaccine target, the TsSP-ZH68 was successfully cloned and expressed in BL21. As shown in **Figure 4A**, the rTsSP-ZH68 was highly induced in *E. coli* BL21 in insoluble fractions. The MW (45.3 kDa) of the rTsSP-ZH68 (with the histidine tag) was identical with the predicted combined size of the protein encoded by TsSP-ZH68 gene and the histidine tag from the plasmid. Western blot analysis showed that the purified rTsSP-ZH68 was recognized by sera of infected mice at 8–10, 15, and 42 dpi, sera of early patients with trichinellosis at 19 and 35 dpi, and anti-His tag mouse monoclonal antibody, but not by sera from normal mice and healthy persons (**Figure 4B**).

## Discussion

In this study, an immunoproteomics approach was used to identify potential early diagnostic antigens or candidate vaccine target for trichinellosis. The AW ES proteins were analyzed by using SDS-PAGE and Western blotting, the five proteins bands (55, 48–50, 45, 44, and 36 kDa) were recognized by sera of early patients with trichinellosis at 19 dpi. The results further demonstrated that the AW ES antigens might be secreted by the parasite into the host’s peripheral blood circulation at early phase of *Trichinella* infection and induced an early specific antibody response (Sun et al., 2015; Liu R.D. et al., 2016). The five protein bands recognized by early infection sera were further characterized by shotgun LC-MS/MS. A total of 185 proteins of *T. spiralis* were identified, and several enzymes (e.g., adult-specific DNase II, serine protease and serine protease inhibitor) could be the invasion-related proteins and early diagnostic antigens for trichinellosis. Then, the identified proteins were further analyzed by GO annotation to provide a comprehensive understand. The identified proteins have a wide diversity of biological functions (binding of nucleotides, proteins, ions, carbohydrates and lipids; activity of hydrolase, transferase and oxidoreductase, lyase, etc.).

The subcategory of transferase activity included phosphotransferase, phosphoglycerate kinase, alpha-(1,3)-fucosyltransferase C, citrate synthase, etc. Fucosylated glycans are produced by fucosyltransferases (FTs), which are a group of enzymes responsible for the transfer of fucose from guanosme 5-diphosphate–a (1–3,4) fucosidase (GDP–Fuc) to oligosaccharide acceptors linked to protein and lipid. Fucosylated glycans play the major roles in the development, survival and adaptation of *Schistosoma mansoni* in its hosts (Trottein et al., 2000; Hokke and Yazdanbakhsh, 2005; Mickum et al., 2016). The recent studies also demonstrated that anti-schistosomal candidate molecules including fucosyltransferases via life cycle transcriptome analyses and gene microarray (Fitzpatrick et al., 2009; Protasio et al., 2012). The subcategory of lyase activity included adenylosuccinate lyase (ASL), diphosphomevalonate decarboxylase and fumarate hydratase class II. ASL is an enzyme in parasite nucleotide salvage pathways that cleaves adenylosuccinate into adenosine 5′-monophosphate and fumarate (Senft et al., 1972; Foulk et al., 2002). In *Leishmania donovani*, ASL is central enzymes in purine salvage and has been validated as a potential drug target (Boitz et al., 2013). Schistosomes rely on purine salvage pathways to obtain nucleotides, so the enzymes of schistosome purine salvage pathways are the molecular targets for anti-schistosomal drugs (Senft et al., 1972; Senft and Crabtree, 1983). In *S. mansoni*, ASL is a crucial enzyme and should be evaluated as a possible chemotherapeutic target (Foulk et al., 2002). In this study, alpha-(1,3)-fucosyltransferase C and ASL was recognized by sera of early patients with trichinellosis, suggesting that they have potential to be targets for vaccine or immunodiagnostic antigens.

In the hydrolase activity subcategory, 60 proteins had hydrolase activity, including serine protease, cytosol aminopeptidase, chymotrypsin-like protease, chymotrypsin-like protease and enteropeptidase, of which the putative serine protease (TsSP-ZH68; uniprot ID: B0F9T9) is of particular interest. The TsSP-ZH68 belongs to peptidase S1A, chymotrypsin family, and chymotrypsin is a digestive enzyme of pancreatic juice in the duodenum where it carries out proteolysis, the breakdown of proteins and polypeptides (Wilcox, 1970). Serine proteases with chymotrypsin-like, elastase-like or trypsin-like activities are the most abundant protease of *T. spiralis* ES products or crude extract (Todorova and Stoyanov, 2000; Wang et al., 2014; Yang et al., 2015b; Liu J.Y. et al., 2016). Serine proteases play a variety of roles during the parasite life cycle, such as parasite development and nutrition, host tissue and cell invasion, anticoagulation, and immune evasion (Yang et al., 2015b). NBL1 is a newborn larva stage-specific serine protease and C-terminal part of NBL1 (NBL1-C) can be useful for the early diagnosis of trichinellosis in pigs, invasion of host cells and protective immune responses during *Trichinella* infection (Yang et al., 2015a, 2016). *T. spiralis* serine protease-1 named TspSP-1 contributes to the movement of the larvae by degrading cytoplasmic or intercellular proteins (Romaris et al., 2002). TspSP-1.2 could play an important role in the larval invasion of host’s intestinal mucosa and might be a potential candidate target for vaccine against *T. spiralis* infection (Wang B. et al., 2013). The 31 kDa proteins screened from *T. spiralis* ML ES proteins by immunoproteomics, belongs to the trypsin-like serine protease superfamily, possess significant potential as early diagnostic antigen for trichinellosis (Cui et al., 2015). The TsSP-ZH68 identified in this study was chosen for expression in recombinant protein in *E. coli* to further evaluate as a reagent for the early serodiagnosis for trichinellosis. The results demonstrated that TsSP-ZH68 is feasible to be expressed as insoluble recombinant protein in *E. coli* and the purified rTsSP-ZH68 from induced bacterial lysates was recognized by sera of infected mice at 8–10 dpi and sera of early patients with trichinellosis at 19 dpi. These results further indicated that TsSP-ZH68 is an immunodominant antigen recognized by sera of early patients with trichinellosis and could be a good diagnostic marker for early trichinellosis. The sensitivity and specificity of rTsSP-ZH68 for detecting anti*-Trichinella* IgG need to be further investigated by using the sera of patients with trichinellosis, other helminthiases and healthy persons.

## Conclusion

In this study, the ES proteins of *T. spiralis* AW were identified and characterized by immunoproteomic analysis with sera of early patients with trichinellosis. A total of 185 proteins in five protein bands (55, 48–50, 45, 44 and 36 kDa) were identified and analyzed by shotgun LC–MS/MS combined with bioinformatics. The AW ES proteins provided a new source of early diagnostic antigens for trichinellosis. The identified proteins (adult-specific DNase II, serine protease and serine protease inhibitor) might be the invasion-related proteins and early diagnostic antigens. The TsSP-ZH68 identified in this study possess significant potential as early serodiagnostic antigen, but its sensitivity and specificity need to be further studied by using sera of patients with trichinellosis and other helminthiases.

## Author Contributions

ZW and JC conceived and designed this study. RL, GS, YS, PJ, and XZ performed the experiments. ZW, RL, and JC wrote and revised the manuscript. All authors approved the final manuscript to be published.

## Conflict of Interest Statement

The authors declare that the research was conducted in the absence of any commercial or financial relationships that could be construed as a potential conflict of interest..

## Footnotes

1 http://www.cbs.dtu.dk/services/SignalP/

2 http://www.cbs.dtu.dk/services/TMHMM/

3 http://wego.genomics.org.cn/cgi-bin/wego/index.pl
